# Prevention of Herpes Zoster: A Focus on the Effectiveness and Safety of Herpes Zoster Vaccines

**DOI:** 10.3390/v14122667

**Published:** 2022-11-29

**Authors:** Yasmin Marra, Fawziah Lalji

**Affiliations:** 1 Faculty of Arts and Sciences, Queen’s University, Kingston, ON K7L 3N6, Canada; 2 Faculty of Pharmaceutical Sciences, University of British Columbia, Vancouver, BC V6T 1Z3, Canada

**Keywords:** herpes zoster, shingles, vaccine effectiveness, zoster vaccine, live attenuated Oka varicella zoster vaccine, recombinant zoster vaccine

## Abstract

Infection with varicella zoster virus typically occurs in children and it can cause primary varicella infection or “chickenpox”, or it can reactivate later in life and cause herpes zoster or “shingles”. Herpes zoster mainly occurs in older adults, causing a reduction in activities of daily living, impacting quality of life, and may lead to serious complications, including chronic pain. Two vaccines are marketed to prevent herpes zoster: the live zoster vaccine and the non-live, recombinant zoster vaccine. The pre-licensure clinical trials show the efficacy of the live zoster vaccine to be between 50 and 70% and for the recombinant vaccine to be higher at 90 to 97%. Real-world effectiveness studies, with a follow-up of approximately 10 years, were reviewed in this article. These data corroborated the efficacy studies, with vaccine effectiveness being 46% and 85% for the live and recombinant vaccines, respectively. Safety data from the effectiveness studies show similar results to the clinical trials with mostly local injection-site reactions and mild systemic reactions seen with both vaccines, although in larger proportions with the recombinant vaccine. Rare adverse events, occurring less than 1% of the time, have been seen with both vaccine types and include disseminated herpes zoster with the live zoster vaccine and Guillain–Barré syndrome with the recombinant vaccine. The wider use of preventative measures with vaccines will reduce the herpes zoster burden of illness seen in older adults.

## 1. Epidemiology of Herpes Zoster and Complications 

Infection with varicella zoster virus (VZV) either causes primary varicella infection (i.e., chickenpox) that typically occurs among children, or herpes zoster (HZ), which is the reactivation of VZV after initial infection (i.e., shingles) [1]. Approximately one in three persons in the general population will develop zoster during their lifetime, and it typically occurs in older adults [2,3]. Herpes zoster manifests as an acute, painful, localized, vesicular rash generally along specific dermatomes [2,4]. Complications of acute zoster are potentially severe and result in significant decreases in patients’ ability to perform their activities of daily living (i.e., quality of life). These include loss of eyesight (zoster ophthalmicus), central nervous system infection, nerve palsies (e.g., Ramsay–Hunt syndrome), and secondary bacterial infections [4,5]. Post-herpetic neuralgia (PHN), which can last for weeks, months, or even years, is the most common complication following a zoster rash. This complication occurs in approximately 20% of adults and is more frequent and severe older patients [2,6].

Many countries worldwide use varicella vaccination for all children aged >12 months to 12 years who lack evidence of immunity. Publicly funded routine varicella immunization programs were implemented between 2000 and 2007, and since that time, vaccination has dramatically reduced the incidence of primary varicella (i.e., chickenpox) among children (a reduction of 97%) [3]. In contrast, many studies from North America, Europe, and Asia-Pacific show that HZ incidence is increasing with time. Using electronic medical record data, administrative data, or surveillance, studies show the HZ incidence rate to range between 3 and 5 per 1000 person-years [7], with the rate increasing over time [8,9,10,11,12]. For example, in one large USA-based study of 27 million persons, the incidence of HZ consultations increased from 1.7 in 1993 to 7.2 per 1000 person-years in 2016 [8]. A cohort study conducted in Canada reported that the incidence of HZ increased from 2.9 per 1000 inhabitants in 1997 to 4.7 per 1000 inhabitants in 2012 [13]. 

An earlier systematic review conducted by Kawai et al. in 2014 reported that HZ incidence increased with advancing age, from 6 to 8 per 1000 person-years at 60 years to 10–12 per 1000 person-years in those aged 80 years and over [7]. A more recent systematic review of five studies reported an HZ incidence of 5 to 8 per 1000 persons in people aged 50 years or over to 11 per 1000 persons in those aged 75 years and over [14]. Age seems to be the biggest risk factor for developing HZ, but a higher incidence is seen in women and those with a family history of zoster disease [7,13,14,15]. 

Primary infection with VZV induces the production of specific memory T-cells that keep the virus from reactivating. Therefore, the factors that decrease cellular immunity, such as aging or conditions/medications that reduce T-cell levels so that they can no longer inhibit viral replication, increase the likelihood of clinical manifestations of the disease [16]. Some of the reasons for the rise in the incidence of HZ include: the aging population and decrease in natural immunity of older adults exacerbating this issue. Secondly, a higher proportion of the general population that is immunocompromised in higher-income countries [7,17,18]. For example, more immunosuppressive medications and biologicals are used to treat autoimmune conditions, leading to decreased immune function. In addition, increased numbers of patients with autoimmune diseases, HIV, cancer, and organ dysfunction that can lead to reduced immunity and increased susceptibility to VZV reactivation. Finally, increased health literacy and awareness of HZ infection may contribute to increased rates of diagnosis. 

## 2. Prevention of Herpes Zoster and Complications: Efficacy and Safety

### 2.1. Zoster Live Vaccine

Herpes zoster is a preventable disease. In 2006, a live, attenuated, one-dose zoster vaccine (ZVL) (ZOSTAVAX^®^, manufactured by Merck & Co., Inc., Rahway, NJ, USA) was licensed for the prevention of zoster in adults aged 60 years and older [19], and revised to adults aged 50 years and older in 2011 [20]. The ZVL vaccine is a more potent version of the chicken pox vaccine (VZV), containing approximately 15-fold more plaque-forming units of the attenuated virus per dose [16]. 

The Shingles Prevention Study was a randomized, multicenter, placebo-controlled, double-blind clinical trial in which 38,546 immunocompetent adults aged ≥60 years received either an Oka/Merck VZV vaccine (zoster vaccine) or a placebo [21]. The primary outcome was not the incidence of HZ, but rather a composite endpoint. The burden of illness due to herpes zoster accounted for the incidence of HZ and total HZ-associated pain, representing the severity and duration of illness. The secondary endpoints were the incidence of HZ, PHN, and impact on health-related quality of life. Table 1 shows that the zoster vaccine reduced the HZ burden of illness by 61.1%, the incidence of HZ by 51.3%, and the incidence of PHN by 66.5% by the end of the trial (median follow-up 3.2 years). The zoster vaccine also reduced the negative impact of the disease on the activities of daily living and health-related quality of life by approximately 50%. Of note, the efficacy of the ZVL for preventing HZ decreased with age such that in the 60–69-year-old age group, the vaccine efficacy was 64%, but decreased to 41% in patients aged 70–79 years, and lower still at 18% in patients ≥80 years. The zoster vaccine was well tolerated, with no differences in the adverse event profile between the vaccine and placebo arms. Based on this landmark clinical trial, most Western countries recommended using the HZ vaccine in those aged 60 years and older.

The second randomized, placebo-controlled, double-blind study was conducted in younger (but still immunocompetent) patients aged 50–59 years (*N* = 22,439) [22]. The patients in the intention-to-treat population were followed for an average of 1.3 years (range, 0 days–2 years) postvaccination and the vaccine efficacy for preventing HZ was 69.8% (Table 1). More adverse events were seen in the HZ arm compared to the placebo arm (ZVL: 73% vs. PLB: 42%), with higher rates of local, injection-site adverse events reported in the vaccine arm. Systemic adverse events occurred equally in the two arms (35% in the vaccine arm and 34% in the placebo arm), with headache being the most commonly reported event. The proportion of patients reporting adverse events occurring within 42 days postvaccination (ZVL: 0.6% vs. PLB: 0.5%) and 182 days postvaccination ZVL: 2.1% vs. PLB: 1.9%) was similar between the two study arms [23]. 

### 2.2. Recombinant Zoster Vaccine

A second HZ vaccine known as the Recombinant Zoster Vaccine (RZV) (Shingrix, GlaxoSmithKline (GSK)) was licensed in 2017 [24]. Unlike ZVL, this is a subunit vaccine containing recombinant glycoprotein E, the main target of CD4+ T-cells, together with an adjuvant (AS01B) to boost the immune response. The vaccine was approved as a two-dose series, administered intramuscularly, 2–6 months apart. The licensing of RZV and subsequent recommendation by national immunization programs in Western countries for use in immunocompetent adults aged ≥50 years was based on two pre-licensure clinical trials: ZOE-50 and ZOE-70 [25,26]. The two trials were similar in design: randomized, placebo-controlled, multicenter studies that evaluated two intramuscular doses of the vaccine or placebo in adults 50 years of age [25] or over and 70 years of age and over [26], respectively. 

The total cohort in ZOE-50 was 15,411 adults aged 50 years and older, stratified by 10-year age strata (50–59, 60–69, and ≥70 years) [25]. The study participants were followed for 60 months, and the primary endpoint was to evaluate its efficacy in reducing the risk of HZ compared to a placebo. Table 2 shows the overall efficacy against HZ, which was 97.2% (95% CI: 93.7 to 99.0) at the mean follow-up of 3.2 years. Unlike the live vaccine that had a lower efficacy in older adults, the RZV efficacy remained high even for the oldest age group, with efficacy being 97% for the youngest group of 50–59 years of age, 97% for those 60–69 years of age, and 91% for individuals 70 years and older. The proportion of adverse events was higher in the vaccine recipients than in the placebo group seven days after vaccination (vaccine arm 84.4% vs. 37.8% placebo arm). Although the majority were of mild-to-moderate intensity, a significant proportion of the vaccine recipients (17% vs. 3%) reported grade 3 symptoms (i.e., symptoms that prevented activities of daily living). These were mostly local reactions (RZV: 81.5% vs. PLB: 11.9%). Grade 3 injection-site events occurred in 9.5% of RZV patients and 0.4% of placebo patients. Systemic reactions were also more common in the RZV patients (RZV: 66.1 (grade 3 in 11.4%) vs. PLB: 29.5% (grade 3 in 2.4%)). Serious adverse events, defined as hospitalization, prolongation of existing hospitalization, life-threatening illness, permanent disability, congenital anomaly/disability, or death, were similar in the two study arms (RZV: 1.1% vs. PLB: 1.3%) in the first 30 days postvaccination. These numbers did not change in the 3.5-year follow-up, with serious adverse events reported as 9% in both study arms. 

ZOE-70 was a similarly designed, randomized, placebo-controlled clinical trial with 13,900 participants who were 70 years or older [26]. The trial used the same endpoints and definitions as ZOE-50, with the primary endpoint reported in the modified vaccinated cohort (i.e., individuals who had received two doses of the vaccine and who did not have an HZ episode within 1 month after the second dose). In this cohort, HZ occurred in 23/6541 vaccinated participants compared to 223/6622 placebo participants during a mean follow-up period of 3.7 years. This translated to a vaccine efficacy of 89.8% (95% CI: 84.2% to 93.7%) and was similar in participants aged 70 to 79 years (90.0%) and participants aged 80 or older (89.1%). The investigators of the two pre-licensure studies conducted a pooled analysis of the participants who were aged 70 years or older (*N* = 16,596) [26]. The vaccine efficacy against HZ was 91.3% (95% CI: 86.8 to 94.5), and vaccine efficacy against PHN was 88.8% (95% CI: 68.7 to 97.1). Furthermore, the vaccine efficacy did not differ significantly when stratified into the two age strata (70–79 years: 91.3% and ≥80 years: 91.4%). 

A total of 1025 participants were assigned to a subgroup to evaluate adverse events—these events were ‘solicited’ [26]. In this subgroup, adverse events within seven days post-immunization were more frequent among the RZV recipients than among the placebo recipients (79.0% vs. 29.5%). Mild, local injection-site reactions (pain, redness, swelling), lasting 2–3 days, occurred in 74.1% of the RZV group compared to 9.9% of the placebo group. Grade 3 injection-site reactions in this subgroup were reported in 8.5% of the RZV group and 0.2% of the placebo group. Systemic solicited adverse events (myalgia, fatigue, headache, fever) also occurred more commonly in the active arm (RZV 53% vs. placebo 25%), with myalgia and fatigue being the most common systemic events, lasting 1–2 days. The overall frequency and severity of the solicited adverse events did not increase significantly with the second dose. The long-term follow-up of adverse events (mean follow-up period of 4.0 years) revealed that the overall incidence of serious adverse events (RZV 16.6% vs. placebo 17.5%) and potential immune-mediated diseases (RZV 1.3% vs. placebo 1.4%) were similar in the two study groups.

## 3. Prevention of Herpes Zoster and Complications: Effectiveness and Safety

Efficacy studies designed as randomized controlled trials investigate the effect and harms of an intervention under tightly controlled conditions, which minimizes bias and gives the studies high internal validity [27]. However, clinical trial data do not always translate to “effectiveness” in the real world [28]. Post-marketing “effectiveness” studies are routinely conducted to evaluate the performance of the vaccine/drug in the heterogeneous population and routine clinical settings, thereby providing external validity.

### 3.1. Zoster Live Vaccine

Numerous post-marketing, observational studies have been conducted over the last two decades to evaluate the effectiveness of ZVL in the general population. These studies have demonstrated comparable vaccine effectiveness (VE) against HZ to that observed in the original Shingles Prevention Study [29]. In addition, a few studies showed that the vaccine effectiveness for the prevention of PHN was higher than the vaccine effectiveness for the prevention of HZ, the vaccine reduced the severity and duration of PHN, and reduced the occurrence of HZO [30,31,32,33]. These reductions are pronounced for patients hospitalized with HZ and complications (i.e., severe cases of HZ) [32]. Tricco et al. conducted the first meta-analysis that evaluated the efficacy, effectiveness, and safety of herpes zoster vaccines against placebo for adults aged 50 years and older [34]. However, it should be noted that the authors included 27 studies (*N* = 2,044,504) in total, with the majority (*N* = 22/27 studies) being efficacy studies. The primary objective was to compare ZVL to placebo or RZV as a network meta-analysis. The network meta-analysis of five randomized controlled trials found no statistically significant differences between ZVL and placebo (ZVL VE: 57%, 95% credible interval −61% to 84%) and ZVL was found to be inferior to RZV (RZV VE: 85%, 95% credible interval 31% to 98%) for the incidence of laboratory-confirmed HZ. 

In contrast, Mbinta and colleagues recently published a systemic review and meta-analysis that only included observational studies (i.e., prospective and retrospective cohort studies and case-control studies [35] (Table 1). The participants were 50 years old or above. They included 22 studies (*N* = 9,536,086 individuals and 3.35 million person-years) that compared the intervention (ZVL or RZV) to no vaccination or another vaccine. Of the 22 studies, one study used a case-control design while the rest were cohort studies; the study duration ranged from 15 months to 198 months. The pooled data from seven studies showed that the vaccine effectiveness for ZVL against herpes zoster in adults was 45.9% (95% CI: 42.2 to 49.4). The vaccine effectiveness for ZVL against PHN was 59.7% (95% CI: 48.3 to 68.7; *N* = 3) and against HZO was 30.0% (95% CI: 20.5 to 38.4; *N* = 2). ZVL effectively prevented HZ in people with comorbidities, such as diabetes, chronic kidney/liver disease, and chronic heart/lung disease with a vaccine effectiveness ranging from 49.8% to 54.3%. Vaccine effectiveness against HZ waned with time, being 60.0% (95% CI: 13.6 to 77.6) during the first year postvaccination and waned to 50.8% (95% CI: 11.4 to 72.9) during the sixth year postvaccination. The authors concluded that ZVL is effective in preventing HZ and PHN in routine clinical practice, although the overall quality of evidence was low and substantial heterogeneity (I2 ≥ 75%) was observed in 50% of the meta-analyses.

In tandem with the effectiveness studies, post-marketing safety studies of ZVL have shown similar results to the original clinical trials. For example, a 10-year surveillance study reported that, after over 34 million doses of ZVL, 93% of the adverse events were non-serious, local, injection-site pain, occurring a median of 2 days postvaccination [36]. The study also noted a rare (<1%) incidence of disseminated HZ; however, when it did occur, 38% of the cases were in immunocompromised individuals. Two recent studies of ZVL safety in Australian adults aged 70–79 years old, one using a self-controlled series design [37] and another using active surveillance [38] to estimate the seasonally adjusted relative incidence of seven prespecified outcome events (injection site reaction, burn, myocardial infarction, stroke, rash, with and without antiviral prescription, and clinical attendance), did not identify any new safety concerns. Three case reports of death from disseminated zoster following the receipt of ZVL have been described in the literature: two of the cases were in persons with chronic lymphocytic leukemia [39,40] and one was in a patient with rheumatoid arthritis (RA) on low-dose corticosteroids and methotrexate [41].

### 3.2. Recombinant Zoster Vaccine

The meta-analysis conducted by Simpson’s team included observational studies between 25 May 2006 to 31 December 2020 [35]; however, the two large RZV effectiveness studies [42,43] and one smaller one [44] was published a year later. Thus, ZVL was the only vaccine type to be examined a priori, and RZV effectiveness was conducted as a post hoc analysis with a pooled vaccine effectiveness of 79.2% (95% CI: 57.6 to 89.7).

Presently, three studies have evaluated RZV vaccine effectiveness (Table S1). All of the studies are similar in design because they are cohort studies using administrative databases that followed vaccinated and unvaccinated cohorts for a median of seven months to look for the incidence of HZ and complications. 

The largest of these studies was conducted by Izurieta and colleagues two years post-licensure [42]. A prospective, cohort study was conducted using Medicare community-dwelling beneficiaries aged 65 years or older with over 15 million beneficiaries. All beneficiaries started as unvaccinated on the index date and switched to the subsequent vaccinated cohorts (either one dose or two doses) within 30 days after vaccine receipt to allow time for an immune response. Since the cohort included community-dwelling residents, nursing home or hospitalized patients were excluded. Of the 15,589,546 beneficiaries, 1,498,275 received one dose of RZV and 1,006,446 received two doses of the vaccine. The individuals in the cohort were followed up until one of the following: receipt of a third dose of the vaccine, termination from insurance coverage, a move to a nursing home, an occurrence of any of the study endpoints (i.e., HZ, HZO, or PHN), or the end of the study period. The vaccinated and unvaccinated individuals in the cohort were compared to determine the vaccine effectiveness for HZ (after the receipt of one or two doses of the vaccine), HZO (after the receipt of two doses), or PHN (after the receipt of two doses). The investigators conducted a subgroup analysis of the vaccine’s effectiveness by different age categories and timing of the second dose beyond 6 months. For the secondary analyses, the investigators evaluated the vaccine effectiveness in those classified as immunocompromised and individuals who had received ZVL five years prior to the index date. For all analyses, they used a marginal structural Cox regression model to estimate the hazard ratios in the vaccinated cohorts compared to the unvaccinated cohort, managing the numerous covariates in the three cohorts (one-dose vaccinated, two-doses vaccinated, and unvaccinated) using the standardized mean difference over time (i.e., inverse probability of treatment weighting). The vaccine effectiveness was estimated as 1-HR × 100%.

The mean age of beneficiaries was 74 years; 78% completed the two doses by 6 months and 86% by 12 months. The adjusted RZV HZ effectiveness after two doses was 70.1% and was 56.9% after one dose, lower than the ZOE-70 clinical trial [26]. The two-dose vaccine effectiveness was not lower for the older age groups (i.e., ≥80 years), with a vaccine effectiveness of 70.6% and 68.5% in the 65–79-year-old group and the over 80-year-old group, respectively. The timing of the second dose did not have an effect on the vaccine effectiveness; the vaccine effectiveness for those who received their second dose within 6 months (median = 90 days) of the first was 70.0% (95% CI: 68.4 to 71.5) compared to vaccine effectiveness of 71.7%; 95% CI: 66.1 to 76.3 in beneficiaries vaccinated after 180 days (median = 230). The vaccine effectiveness against PHN was comparable to HZ (VE 76.0% (95% CI: 68.4 to 81.8), but was lower for HZO at 66.8% (95% CI: 60.7 to 72.0). The ZOE-70 clinical trial excluded immunocompromised individuals; however, Izurieta et al. evaluated the effectiveness against immunocompromised and non-immunocompromised beneficiaries and reported their vaccine effectiveness as 64.1% vs. 70.9%, respectively, which is a modest difference. Finally, the authors evaluated the vaccine effectiveness among beneficiaries who had previously received ZVL compared to those who had not, and showed an RZV vaccine effectiveness of 63%; the authors suggested that this low vaccine effectiveness was due to the residual protection offered by the previous ZVL vaccination among the RZV-unvaccinated group. 

In contrast to the previous study, Sun et al. provided evidence of RZV effectiveness among enrollees aged 50 and older in the United States [43]. This was a retrospective cohort design with beneficiaries enrolled in a commercial, Medicare Part D, and Medicare Advantage databases. A total of 4,769,819 patients, 3.6% (*N* = 173,745) of whom received two doses of RZV, were followed for a median of 7 months to evaluate the vaccine effectiveness for two doses of RZV. The patients were followed from the index date until one of the following events: HZ diagnosis, the development of immunocompromised status, ZVL receipt, disenrollment from the insurance plan, or the end of the study period. Cox proportional hazards regression models were used to estimate the HR associated with RZV, and inverse probability weighting was used to control for confounding. The median age of the patients was 65 years, and the adjusted vaccine effectiveness in the RZV recipients aged 50–79 years was 85.5%, which was higher than Izurieta’s study [42]. Of note, the RZV vaccine effectiveness of 85.5% was lower than the ZOE-50 clinical trial that had an overall efficacy between 96.6% and 97.9% among all age groups from 50 years and above [25]. Sun et al. found the vaccine effectiveness in recipients ≥80 years old to be 80.2%, compared with 89.8% in ZOE-70 [26]. No differences in the vaccine effectiveness were seen according to sex or race/ethnicity. Patients with a history of live zoster vaccine within 5 years of the study inclusion retained modest protection, but the addition of the RZV vaccine gave added protection with an effectiveness of 84.8%. Sun et al. published another study with the same methodology, but using the Hawaii Kaiser Permanente database [44]. A total of 78,356 adults aged 50 years or older were included with a median age of 61 years (IQR: 54–69) [43]. The vaccine effectiveness was shown to be 83.5% against HZ and was high at 93.3% against HZO, but the sample size in the latter group was very small, resulting in a large confidence interval (PHN was not assessed in this study).

The lower vaccine effectiveness in the real-world studies compared to the ZOE-50 [25] and ZOE-70 [26] clinical trials may be due to any number of factors, despite the authors doing their best to adjust for some of these in their analyses (Table 2) [45,46]. Differences in the study population demographics (age, sex, race/ethnicity), the presence of comorbid conditions, including being immunocompromised (the clinical trials favored mostly healthy adults), the uptake rate of the vaccine, timing of the vaccine, health-seeking behavior [47], diagnosis, coding issues, and residual confounding can all impact vaccine effectiveness. For example, in the clinical trials, case finding was active, but in these observational studies, only those who attended medical facilities were included, leading to misclassification bias. Although the positive predictive value of HZ coding is high, observational studies will pick up miscoding (e.g., false positives), diluting the VE estimates. All three observational studies balanced their cohorts using inverse probability weighting, but imbalances remained in the different cohorts, giving rise to residual confounding that would impact VE estimates. 

After licensure in the USA, the Centers for Disease Control and Prevention (CDC) and the Food and Drug Administration (FDA) began the safety monitoring of RZV in the Vaccine Adverse Event Reporting System (VAERS), a USA passive surveillance system for adverse events that occur from immunizations [48]. The report, published in 2019 and based on 3.2 million doses of RZV, found 4381 reports of adverse events [49] and a subsequent 2020 study found similar results [50]. The commonly reported signs and symptoms were consistent with the safety profile observed in prelicensure clinical trials, and included fever (23.6%), injection site pain (22.5%), and injection site erythema (20.1%). Adverse events categorized as serious occurred in 3% of the population [49]. All of the adverse events reported for RZV were similar to the proportions observed for other vaccines in the VAERS database. The adverse events were also similar to the frequencies reported in the two landmark clinical trials in which 85% of 6773 vaccinated study participants reported local or systemic reactions after receiving RZV, with approximately 17% experiencing a grade 3 reaction. However, the rates of serious adverse events were lower in the clinical trials, at approximately 1% or less, in both the RZV and placebo groups; this difference may be related to a smaller sample size in the trials [49].

Using a database designed to pick up serious and rare safety signals, the Vaccine Safety Datalink (VSD), the CDC conducted a post-marketing safety study among individuals aged 50 years and older who had received RZV [51]. The VSD analyses identified a preliminary statistical signal suggesting an increased risk of Bell’s palsy (RR = 1.51) and GBS (RR + 5.25) among individuals who received RZV compared to ZVL recipients (historical controls). The Bell’s palsy effect did not persist as more doses accrued (RR = 0.90), and although the GBS signal waned with time (RR = 1.24), it remained statistically significant. To evaluate this statistical signal further, the investigators used a self-controlled case series design among individuals aged 65 years or older [52]. Over a period of roughly three years, 2,113,758 Medicare beneficiaries who had received 3,729,863 vaccinations (due to the two-dose schedule) were identified and followed for GBS-related hospitalization. The relative risk of Guillain–Barré syndrome after RZV vs. ZVL was compared during a postvaccination risk window of 1–42 days and a control window of 43–183 days. The investigators identified an attributable risk of 3.13 (95% CI: 0.62–5.64) excess cases per million doses administered within the first 42 days postvaccination. In the secondary analyses, the investigators evaluated the risk according to the number of doses and found that the second dose did not increase the GBS risk further. The FDA and the CDC’s Advisory Committee on Immunization Practices (ACIP) carefully considered the results of this study. They acknowledged an association of GBS with RZV, and that this adverse event has also been associated with other vaccines [53,54], but felt the available evidence did not establish a causal link for the recombinant vaccine. Although the FDA asked for a warning to be placed on the product monograph [55], the ACIP did not change their recommendations for RZV vaccination, citing that the public health benefits of RZV in preventing herpes zoster overcome the small attributable risk of GBS, and undertaking a formal risk–benefit analysis [32]. 

## 4. Duration of Protection of Herpes Zoster Vaccines

### 4.1. Zoster Live Vaccine

The duration of protection for ZVL has been studied up to 11 years (4 years follow-up of RCT patients, followed by the open-labelled arm from years 7 to 11) [21,33,56]. Consistent results have been shown in the long-term follow-up RCT and observational studies that suggest a steep decline in effectiveness following the first year after the receipt of ZVL, and, by 6 years postvaccination, the vaccine effectiveness against HZ is less than 35% and less than 45% for PHN [54,57,58,59]. During years 7–8 postvaccination, the observational study estimates of effectiveness ranged from 21% to 32% [32,33]. Morrison and colleagues had the longest follow-up period, which was 11 years postvaccination, and in their study, the vaccine effectiveness estimates were no longer statistically significant 9 to 11 years after vaccination [55]. This rapid decline in the effectiveness of the HZ vaccine suggests a revaccination strategy would be needed for ZVL recipients [17].

### 4.2. Recombinant Zoster Vaccine 

Boutry et al. followed up 7413 participants enrolled in the long-term follow-up study of the ZOE-50 and ZOE-70 clinical trials. These patients received two doses of RZV and were followed for approximately 7 years. The RZV vaccine efficacy remained over 84% for the duration of the study and did not wane significantly with time. In this cohort of patients, the vaccine efficacy was 97.7% during year 1, dropping slightly by year 3 to 92.4%, and was down to 84.1% during year 8 after the second vaccination [60]. This slow decay was corroborated by the results of the geometric mean concentrations of antibody titers and CD4 T-cells that reached a plateau at approximately six-fold above pre-vaccination levels. The slow waning of immunity would suggest two doses of RZV likely confers long-term immunity and further booster doses may not be needed with this vaccine. 

## 5. Conclusions

The largest risk factor for the development of HZ is age, and the world’s population is aging, with a doubling of older adults projected between now and 2050. Maintaining a healthy, independent older population is paramount to reduce the burden on our healthcare systems. HZ has a major impact on adults, with long-standing consequences in terms of chronic pain, reduction in activities of daily living, and quality of life. Prevention is a key strategy against HZ and for this, the use of either ZVL or RZV is paramount. Since the vaccine effectiveness for ZVL is lower than for RZV, the waning of immunity occurs faster, and there are challenges around its use in immunocompromised populations, many countries are preferentially recommending two doses of RZV in adults aged 50 years and older. Real-world data from effectiveness studies corroborate the evidence seen in the clinical trials for the RZV vaccine, with a vaccine effectiveness of approximately 85% that is maintained for close to ten years. The vaccine safety for RZV is excellent, with mostly local or minor systemic adverse events seen in the majority of the patients, although the small safety signal of Guillain–Barré syndrome needs to be continuously evaluated.

## Figures and Tables

**Table 1:** Efficacy and effectiveness of the live herpes zoster vaccine (ZVL) in adults.

	Efficacy		Effectiveness ^1^	
Author, Year	Oxman (2005)	Schumader (2012)	Tricco (2018)	Mbinta (2022)
Age, years	≥60	≥50	≥50	≥50
Efficacy against HZ, % (95% CI)				
All patients	51.3 (44.2–57.6)	69.8 (54.1–80.6)	57.0 (−61.0–84.0)	45.9 (42.2–49.4)
50–59 yrs	-----	69.8 (54.1–80.6)	-----	60.0 (53.0–65.0)
60–69 yrs	63.9 (N/A)	-----	-----	50.9 (45.0–56.1)
70–79 yrs	37.6 (N/A)	-----	-----	46.6 (40.7–51.9)
≥80	18.0 (N/A)	-----	-----	43.9 (37.7–49.5)
Efficacy against PHN, % (95% CI)				
All patients	66.5 (47.5–79.2)	-----	-----	59.7% (48.3–68.7)
50–59 yrs	-----	-----	-----	-----
60–69 yrs	65.7 (20.4–86.7)	-----	-----	-----
70–79 yrs	66.8 (43.3–81.3)	-----	-----	-----
≥80	-----	-----	-----	-----

^1^ Pooled vaccine effectiveness. Abbreviations: HZ—herpes zoster; PHN—post-herpetic neuralgia; CI—confidence interval.

**Table 2:** Efficacy and effectiveness of the recombinant zoster vaccine (RZV) in adults.

	Efficacy		Effectiveness ^1^	
Author, Year	Lal ZOE-50 (2015)	Cunningham ZOE-70 (2016)	Izurieta (2021)	Sun (2021)
Age, years	≥50	≥70	≥65	≥50
Efficacy against HZ, % (95% CI)	97.2 (93.7–99.0)	89.8 (84.2–93.7)	70.1(68.6–71.5)	68.3 (64.4–71.7)
50–59 yrs	96.6 (89.6–99.3)	-----	-----	85.6 (53.3–95.6)
60–69 yrs	97.4 (90.1–99.7)	-----	70.6 (68.9–72.1) ^2^	87.7 (82.5–91.4)
70–79 yrs	97.9 (87.9–100)	90.0 (83.5–94.4)	70.6 (68.9–72.1) ^2^	86.5 (83.9–88.6)
≥80	-----	89.1 (74.6–96.2)	68.5 (65.1–71.6)	80.3 (75.1–84.3)
Efficacy against PHN, % (95% CI)				
50–59 yrs	91.2 (75.9–97.7) ^1^	91.2 (75.9–97.7) ^1^	76.0 (68.4–81.8)	-----
60–69 yrs	-----	-----	-----	-----
≥70 yrs	88.8 (68.7–97.1)	88.8 (68.7–97.1)	-----	-----
Efficacy against HZO, % (95% CI)	-----	-----	66.8 (60.7–72.0)	-----

^1^ Pooled vaccine effectiveness for PHN from two clinical trials. ^2^ Vaccine effectiveness estimates for individuals aged 65 to 79 years. Abbreviations: HZ—herpes zoster; PHN—post-herpetic neuralgia; HZO—herpes zoster ophthalmitis; CI—confidence interval.

## Supplementary Materials

The following supporting information can be downloaded at: https://www.mdpi.com/article/10.3390/v14122667/s1, Table S1: Study design and results of recombinant zoster vaccine effectiveness studies.
