# Supplementary material for: Prevention of Herpes Zoster: A Focus on the Effectiveness and Safety of Herpes Zoster Vaccines
Source: Viruses. 2022 Nov 29;14(12):2667. doi: 10.3390/v14122667 (PMC9782228; doi:10.3390/v14122667)
Supplement: Supplementary file 1 [file viruses-14-02667-s001.zip › viruses-2050315-supplementary.pdf]

Table S1. Study design and results of recombinant zoster vaccine effectiveness studies

	**Izurieta**	**Sun**	**Sun**
Publication, Year	Clin Infect Dis, 2021	Clin Infect Dis 2021	Vaccine 2021
Design	Prospective, cohort	Retrospective, cohort	Retrospective, cohort
Data Source, Country	Medicare administrative data claims, USA	OptumLabs Data Warehouse claims database (enrollees in Medicare Advantage or Medicare Part D), USA	Kaiser Permanente claims database (enrollees in Medicare Advantage or Medicare Part D), USA
Study Period	November 1, 2017 to October 20, 2019	January 1, 2018 to December 31, 2019	January 1, 2018 to December 31, 2019
Population	Community-dwelling older adults (≥65 y), continuously enrolled in Medicare plan for 12-15 months prior to index date; not in NH/LTCF; no diagnosis of HZ for 12 months prior to index date	Patients (≥50 y), with at least 12 months of continuous enrollment in healthcare plan prior to the index date	Patients (≥50 y), with at least 12 months of continuous enrollment in healthcare plan prior to the index date
Excluded		Individuals who were classified as immunocompromised within 1 year of the index date<br><br>ICD9/10 codes for HZ within 1 year of index date<br><br>Patients with only a single dose of RZV.<br><br>Patients with 2 doses but the first dose was before the index date<br><br>Patients with HZ ≤30 days after second dose of RZV<br><br>Patients who had any HZ diagnosis code between 1st and 2nd dose	Individuals who were classified as immunocompromised within 1 year of the index date<br><br>ICD9/10 codes for HZ within 1 year of index date<br><br>Patients with only a single dose of RZV.<br><br>Patients with 2 doses but the first dose was before the index date<br><br>Patients with HZ ≤30 days after second dose of RZV<br><br>Patients who had any HZ diagnosis code between 1st and 2nd dose

Follow-up	Until occurrence of any of the following:<br>a subsequent claim for a third dose of RZV; death; termination from Medicare coverage; admission to a NH/LTCF; occurrence of either HZ, HZO, or PHN; or end of the study period.	Until occurrence of any of the following:<br>HZ diagnosis, development of immunocompromised status, ZVL receipt, disenrollment from the insurance plan, or end of the study period (31 December 2019).	Until occurrence of any of the following:<br>HZ diagnosis, development of immunocompromised status, ZVL receipt, disenrollment from the insurance plan, or end of the study period (31 December 2019).
HZ definition	Incident HZ as outpatient diagnosis using ICD10 codes (B020, B021, B022.x, B027-B029) in any position<br><br>And<br><br>A claim for HZ-specific antiviral within 7 days of diagnosis	Incident HZ in any clinical setting (outpatient, inpatient, ER, NH) diagnosis using ICD9 code (053.xx;) or ICD10 code (B02.xx) in any position	Incident HZ in any clinical setting (outpatient, inpatient, ER, NH) diagnosis using ICD9 code (053.xx;) or ICD10 code (B02.xx) in any position
HZO/PHN definition	HZO: ICD10 code (B023.x) plus antivirals within 7 days of index date and<br>PHN: ICD-10 B02.2X<br><br>PHN within 90-180 days after HZ onset.	HZO: N/A<br>PHN: N/A	HZO: ICD10 code (B023.x)<br>PHN: N/A
Covariates	Age, sex, race, immunocompromised, chronic conditions, frailty and functional characteristics, healthcare utilization (inpatient stay, long-term care, emergency department, outpatient hospital, and office visits), preventative services utilization.	Age, sex, race, ethnicity, healthcare utilization (inpatient stay, long-term care, emergency department, outpatient hospital, and office visits), age adjusted Charlson comorbidity index, and systemic antiviral use.	Age, sex, race, ethnicity, healthcare utilization (inpatient stay, long-term care, emergency department, outpatient hospital, and office visits), age adjusted Deyo comorbidity index, and systemic antiviral use.
Primary Analyses	VE for HZ (1 and 2 doses)<br>VE for HZO/PHN (2 doses)<br>VE for HZ by age group<br>VE for HZ by timing of the second dose	VE for HZ (2 doses)	VE for HZ (2 doses)

Secondary Analyses	VE for HZ by IC status<br>VE for HZ by ZVL vaccination status<br>VE in individuals with selected conditions	VE for HZ by age group<br>VE for HZ by ZVL vaccination status	VE for HZO (2 doses)<br>VE for HZ by age group<br>VE for HZ by ZVL vaccination status
Number of Patients	Unvaccinated = 13,084,825<br>Vaccinated (1 dose) = 1,498,275<br>Vaccinated (2 doses) = 1,006,446<br>Total = 15,589,546	Unvaccinated = 4,596,074<br>Vaccinated (1 dose) = N/A<br>Vaccinated (2 doses) = 173,745<br>Total = 4,769,819	Unvaccinated = 66,492<br>Vaccinated (1 dose) = N/A<br>Vaccinated (2 doses) = 11,864<br>Total = 78,356
VE for HZ with 1 dose (95% CI)	56.9<br>(55.0-58.8)	N/A	N/A
VE for HZ with 2 doses (95% CI)	70.1<br>(68.6-71.5)	68.3<br>(64.4-71.7)	83.5<br>(74.9-89.2)
VE for HZ (2 doses) by age (95% CI)	<u>Age: 50-64 years</u><br>N/A<br><br><u>Age: 65-79 years</u><br>70.6 (68.9-72.1)<br><br><u>Age: 80+ years</u><br>68.5 (65.1-71.6)	<u>Age: 50-59 years</u><br>85.6 (53.3-95.6)<br><br><u>Age: 60-69 years</u><br>87.7 (82.5-91.4)<br><br><u>Age: 70-79 years</u><br>86.5 (83.9-88.6)<br><br><u>Age: 80+ years</u><br>80.3 (75.1-84.3)	<u>Age: 50-59 years</u><br>100<br><br><u>Age: 60-69 years</u><br>67.7 (11.8-88.1)<br><br><u>Age: 70-79 years</u><br>83.3 (70.1-90.7)<br><br><u>Age: 80+ years</u><br>86.4 (73.5-93.0)
VE for HZ by timing of dose #2 (95% CI)	<u>Given ≤180days</u><br>70.0 (68.4-71.5)<br><br><u>Given >180days</u><br>71.7 (66.1-76.3)	N/A	N/A
VE for HZ by IC status after 2 doses (95% CI)	<u>Not Immunocompromised</u><br>70.5 (69.0-72.0)<br><br><u>Immunocompromised</u><br>64.1 (57.2-69.8)	N/A	N/A
VE for HZO after 2 doses (95% CI)	66.8 (60.7-72.0)	N/A	93.3 (48.7-99.1)

VE for PHN after 2 doses (95% CI)	76.0 (68.4-81.8)	N/A	N/A
VE in those with history of ZVL in the prior 5 years (95% CI)	History of ZVL<br>63.0 (58.3-67.2)	No History of ZVL<br>87.1 (83.4-89.9)<br><br>History of ZVL<br>84.8 (75.3-90.7)	No History of ZVL<br>61.1 (-124.9-93.3)<br><br>History of ZVL<br>83.9 (75.2-89.5)

Abbreviations: HZ – herpes zoster; PHN – post-herpectic neuralgia; HZO herpes zoster ophthalmicus; ICD International Classification of Diseases; HR hazard ratio, CI confidence interval; VE vaccine effectiveness (adjusted); RZV recombinant zoster vaccine; ZVL zoster vaccine live; IC – immunocompromised
